# Directed Differentiation of Human Pluripotent Stem Cells to Podocytes under Defined Conditions

**DOI:** 10.1038/s41598-019-39504-8

**Published:** 2019-02-26

**Authors:** Tongcheng Qian, Shaenah E. Hernday, Xiaoping Bao, William R. Olson, Sarah E. Panzer, Eric V. Shusta, Sean P. Palecek

**Affiliations:** 1 Department of Chemical & Biological Engineering, University of Wisconsin, Madison, WI 53706 USA; 2 School of Medicine and Public Health, University of Wisconsin, Madison, WI 53706 USA

## Abstract

A major cause of chronic kidney disease (CKD) is glomerular disease, which can be attributed to a spectrum of podocyte disorders. Podocytes are non-proliferative, terminally differentiated cells. Thus, the limited supply of primary podocytes impedes CKD research. Differentiation of human pluripotent stem cells (hPSCs) into podocytes has the potential to produce podocytes for disease modeling, drug screening, and cell therapies. In the podocyte differentiation process described here, hPSCs are first induced to primitive streak-like cells by activating canonical Wnt signaling. Next, these cells progress to mesoderm precursors, proliferative nephron progenitors, and eventually become mature podocytes by culturing in a serum-free medium. Podocytes generated via this protocol adopt podocyte morphology, express canonical podocyte markers, and exhibit podocyte phenotypes, including albumin uptake and TGF-β1 triggered cell death. This study provides a simple, defined strategy to generate podocytes for *in vitro* modeling of podocyte development and disease or for cell therapies.

## Introduction

Podocytes are specialized cells that wrap around the capillaries of the glomerulus and comprise the major filtration barrier in the kidney^1^. The slit diaphragm serves as a size-selective macromolecular sieve, allowing water-soluble small molecules to pass through but retaining proteins and large molecules^2,3^. The permeability of slit diaphragms in the glomerular filtration barrier is regulated by tight junction proteins and specialized slit diaphragm proteins, including P-cadherin, occludin, ZO-1, nephrin, podocin, and CD2AP in podocyte foot processes^4^. Dysfunction of glomeruli and the associated loss of filtration capacity is the major cause of end-stage renal diseases^5–7^. Many diseases, including autoimmune disorders^8–10^, bacterial endocarditis^11^, HIV^12^, Alport syndrome^13^, diabetes^14^ and hypertension^15^, affect kidney function by disrupting the function of the glomeruli. The “podocyte depletion” hypothesis describes the mechanism by which initial podocyte injury, which may arise from various cytotoxic, genetic, hemodynamic, infectious, oxidative, or immune insults, leads to loss of podocytes, altered glomerular structure and function, progressive glomerulosclerosis, decline in kidney function, and eventual development of end-stage renal disease (ESRD)^16^. In the United States, approximately 30 million people have chronic kidney disease (CKD) and nearly 1 million patients have been diagnosed with ESRD^17^. The majority of the patients with ESRD are treated with dialysis instead of kidney transplantation^18^ because of an insufficient supply of transplantable organs.

Podocytes are terminally differentiated cells^19^, and currently there is no replacement for dysfunctional podocytes. Immortalized human podocytes have been used to study mechanisms of glomerular diseases^19–21^. However, primary and immortalized podocytes tend to dedifferentiate *in vitro*, thereby losing cobblestone morphology, displaying reduced expression of synaptopodin, and exhibiting unstructured and dysfunctional slit diaphragms^22^. Primary animal podocytes are also used to study the role of podocytes in normal kidney function and glomerular diseases^23,24^, but due to the species differences these animal models fail to recapitulate the functional, structural and molecular aspects of human podocytes^25^. Thus, a source of human podocytes exhibiting key phenotypes of healthy and diseased podocytes would be a valuable tool for advancing kidney research and developing treatments for CKD. Because of their unlimited self-renewal capacity and ability to differentiate into any cell type, human pluripotent stem cells (hPSCs) are a promising source of human podocytes. Recently, several studies showed that hPSCs can generate renal lineages and kidney organoids^26–31^, and podocytes have also been differentiated from hPSCs via activation of Wnt and BMP signaling^32–35^. Here we report a simple method to differentiate hPSCs into podocytes that only requires small molecule activation of Wnt signaling and subsequent culture in podocyte permissive medium. Using this method, hPSCs are first directed to primitive streak-like cells and intermediate mesoderm, then progress to proliferative nephron progenitors and finally differentiate to mature podocytes that express key podocyte-related proteins, including PAX2, WT1, nephrin and synaptopodin. These hPSC-derived podocytes also exhibit podocyte phenotypes, including albumin uptake and induction of cell death upon TGF-β1 stimulation. This differentiation method has the potential to generate podocytes for disease modeling, drug screening, and development of podocyte cell therapies.

## Results

### Wnt activation directs hPSCs to primitive streak and intermediate mesoderm

**Figure 1:**
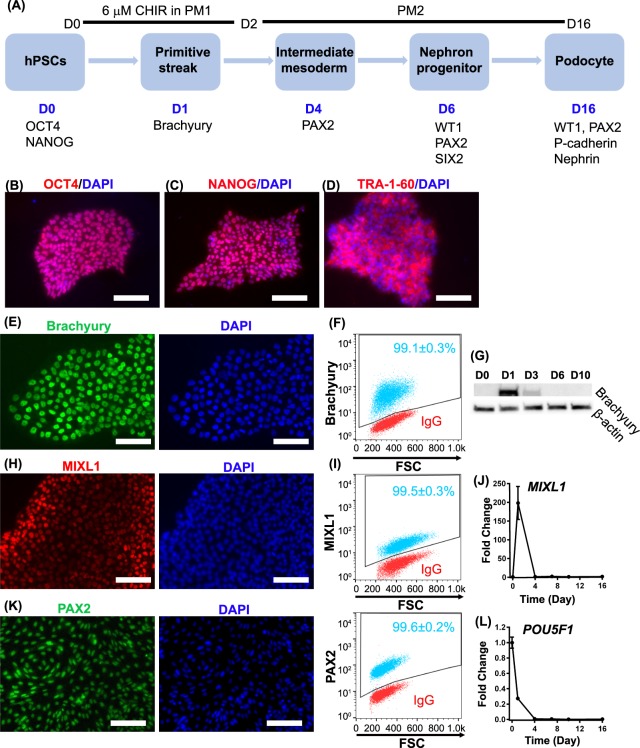
Schematic of podocyte differentiation protocol. (**A**) Before differentiation, singularized IMR90-4 iPSCs are seeded on 12-well plates coated with Matrigel, vitronectin or Synthemax at 2 × 10^4^ cells/cm^2^ and expanded for 3 days in mTeSR1. Differentiation to primitive streak-like cells is initiated by 48 hr treatment with 6 µM CHIR99021 in podocyte medium 1 (PM1). Cells progress to nephron progenitors at day 6 and eventually differentiate to podocytes in podocyte medium 2 (PM2) at day 16. The pluripotent state of expanded IMR90-4 iPSCs was verified prior to differentiation by immunofluorescence for (**B**) OCT4, (**C**) NANOG and (**D**) TRA1-60. Expression of brachyury during differentiation was assessed by (**E**) immunofluorescence and (**F**) flow cytometry 24 hr after CHIR99021 treatment, and (**G**) Western blot from day 0 to day 16. Expression of primitive streak marker MIXL1 during differentiation was assessed by (**H**) immunofluorescence and (**I**) flow cytometry. Expression levels of primitive streak gene *MIXL1* (**J**) and the pluripotency gene *POU5F1* (**L**) relative to the housekeeping gene *GAPDH* were assessed by qRT-PCR from day 0 to day 16. Expression levels were normalized to undifferentiated IMR90-4 iPSCs at day 0. (**K**) At day 4, expression of the intermediate mesoderm marker PAX2 was assessed by immunofluorescence and flow cytometry. In flow cytometry plots, red dots represent isotype control treated cells used to identify the gated regions and blue dots represent cells stained for the indicated marker. Numbers indicate the fraction of stained cells (blue) in the gated regions. Data were collected from three independent replicates and are plotted as mean ± SEM. Scale bars, 100 µm. Immunofluorescence labelling and flow cytometry were performed ten times from different differentiations on different days. Three technical replicates were used each time for flow cytometry. qPCR was performed three replicates each time and three times were performed from three different differentiations.

Most cells forming the kidney, including podocytes, originate from intermediate mesoderm^36^. Wnt signaling plays an important role in the development of mesodermal lineages and induction of canonical Wnt signaling has been used to direct hPSC differentiation to mesoderm^37–39^. For example, in prior studies we showed that treatment of undifferentiated hPSCs with 6 µM CHIR99021, a GSK3β inhibitor, in a serum-free and albumin-free medium generated a uniform population of cells expressing primitive streak markers after 24 hr^40,41^. Thus, as a first step toward differentiating hPSCs to podocytes, IMR90-4 induced pluripotent stem cells (iPSCs) were seeded on a Matrigel-coated surface at a density of ~2 × 10^4^ cells/cm^2^ in mTeSR1. After 3 days of expansion to a density of 6 × 10^4^ cells/cm^2^, we treated the iPSCs with 6 µM CHIR99021 for 48 hr in serum-free and albumin-free podocyte medium 1 (PM1: DMEM/F12, 1% MEM-NEAA, 0.5% GlutaMAX, 0.1 mM β-mercaptoethanol) (Fig. 1A). Before initiating differentiation at day 0, expression of pluripotency markers OCT4, NANOG, and TRA-1-60 was validated by immunofluorescence (Fig. 1B–D). After 24 hr of treatment with CHIR99021, the cells uniformly expressed the primitive streak marker brachyury, which localized to the nucleus in nearly 100% of the cells (Fig. 1E, F). As expected, brachyury expression was transient, disappearing after day 3 (Fig. 1G). After 48 hr of CHIR99021 treatment in PM1, the medium was switched to serum-free podocyte medium 2 (PM2: human endothelial serum-free medium (hESFM), 2% B27 supplement) and cultured to day 16. We further assessed the kinetics of primitive streak induction by immunofluorescence and qRT-PCR. The primitive streak gene *MIXL1* reached peak expression at 24 hr and then was undetectable after day 4 (Fig. 1H, I, J). At day 4, nearly 100% of cells expressed the intermediate mesoderm marker PAX2, which localized to the nucleus (Fig. 1K). Expression of the pluripotency gene *POU5F1* dramatically decreased after initiation of differentiation (Fig. 1L).

### Primitive streak cells progress to nephron progenitors

**Figure 2:**
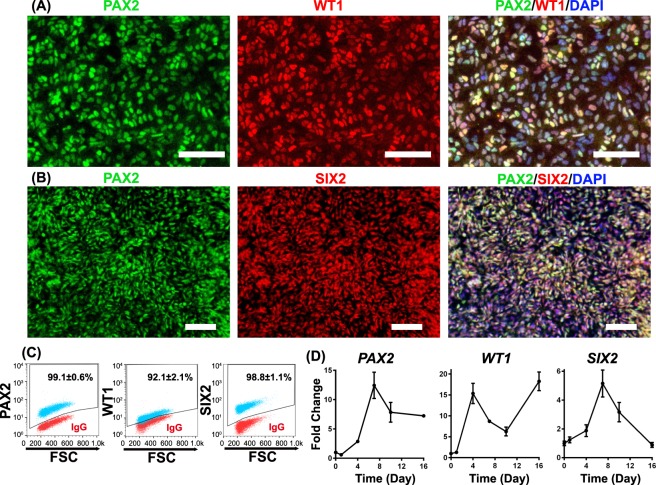
Cells progress to nephron progenitors at Day 6. At differentiation day 6, cells were assessed by immunofluorescence and flow cytometry for nephron progenitor markers, including PAX2 and WT1 (**A**, **C**), and SIX2 (**B**, **C**). In (**C**), red dots represent isotype control treated cells used to identify the gated regions and blue dots represent cells stained for the indicated marker. Numbers indicate the fraction of stained cells (blue) in the gated regions. (**D**) Quantitative RT-PCR was used to monitor the expression of *PAX2*, *WT1*, *SIX2* relative to the housekeeping gene *GAPDH* during IMR90-4 iPSC differentiation to podocytes using the protocol illustrated in Fig. 1A. Expression levels were normalized to undifferentiated IMR90-4 iPSCs. Data are presented as mean ± SEM of three independent experiments. Scale bars, 100 µm. Immunofluorescence labelling and flow cytometry were performed ten times from different differentiations on different days. Three technical replicates were used each time for flow cytometry. qPCR was performed three replicates each time and three times were performed from three different differentiations.

At day 6, immunofluorescence analysis indicated that the differentiating cells possessed nuclear localization of nephron progenitor proteins PAX2, WT1, and SIX2 (Fig. 2A, B). Both PAX2 and WT1 are expressed in nephron progenitors and mature podocytes, while SIX2 is transiently expressed in nephron progenitors^42^. At day 6, over 90% of the cells expressed PAX2, WT1, and SIX2, as determined by flow cytometry (Fig. 2C). By qRT-PCR, *PAX2* and *WT1* expression gradually increased through day 5 and remained expressed through day 16 of differentiation. *SIX2* expression peaked at day 7 and then decreased afterwards (Fig. 2D). However, we did not observe expression of the metanephric marker HOXD11 by immunostaining or RT-qPCR in these cells (data not shown), which might indicate that HOXD11 is not induced during differentiation in this method. HOXD11 knockout does not dramatically impair kidney development in the mouse^43,44^; Other HOX11 paralogs may be involved in specifying nephron progenitors in this podocyte differentiation method.

**Figure 3:**
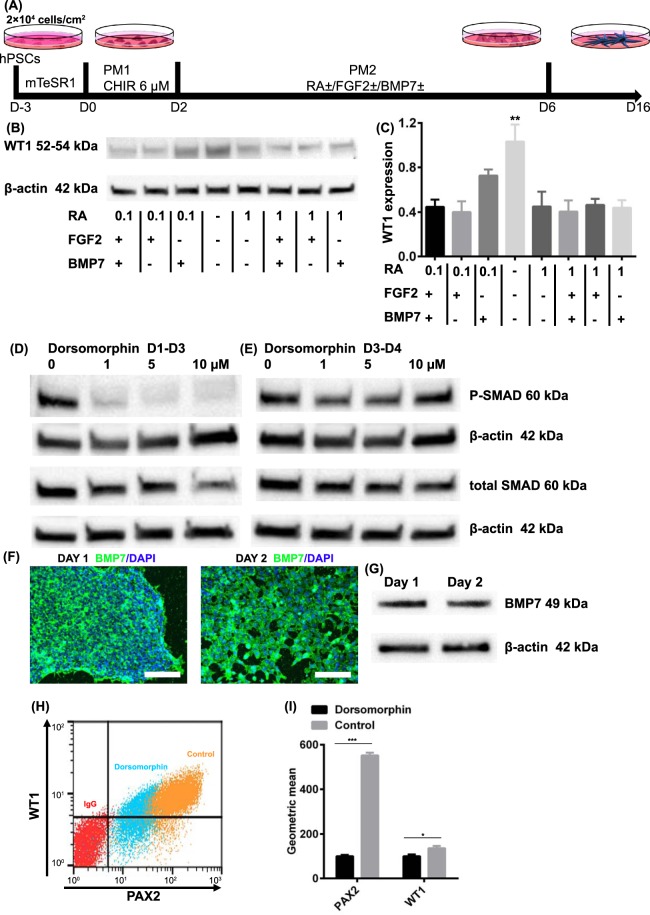
Wnt signaling activation directs hPSCs to nephron progenitors. (**A**) Schematic of podocyte differentiation factor identification. Singularized IMR90-4 iPSCs were seeded on 12-well plates coated with Matrigel and expanded for 3 days in mTeSR1. Podocyte differentiation was initiated with treatment of 6 µM CHIR for 48 h in PM1 followed by treatment with different combinations of 0.1 µM or 1 µM RA, 50 ng/mL BMP7 and 50 ng/mL FGF2 from day 2 to day 6 in PM2. (**B**) Cells at day 6 were assessed by Western blot for the expression of WT1. (**C**) Expression level was quantified via Image J. The expression was normalized to β-actin and then compared to WT1 expression in the absence of FGF2, RA and BMP7. (**D, E**) SMAD phosphorylation was assessed by Western blot after cells were treated with dorsomorphin (0 µM to 10 µM) from day 1 to day 3 or from day 3 to day 4. (**F**, **G**) Expression of BMP7 at days 1 and 2 was verified by immunofluorescence and Western blot of cell lysate (**H**, **I**) Cells were differentiated in the presence or absence of 1 µM dorsomorphin treatment from day 1 to day 3 and at day 16 were assessed for expression of PAX2 and WT1 by flow cytometry. Data were collected from three independent replicates and are plotted as mean ± SEM. *p < 0.05. ***p < 0.001. Scale bars, 100 µm. Immunofluorescence labelling and flow cytometry were performed ten times from different differentiations on different days. Western blot was performed three times from three different differentiations.

Although we observed cells expressing podocyte markers in the growth factor-free PM2 medium, BMP4 and BMP7^32,45,46^, retinoic acid (RA)^47,48^, and FGF2^49^ have previously been shown to be involved in kidney development. Thus, we examined whether addition of these factors would alter the conversion of iPSCs to nephron progenitors. After 2 days of canonical Wnt pathway activation by CHIR99021, we treated cells with combinations of BMP7, RA, and FGF2 from day 2 to day 6 (Fig. 3A) in PM2 and assessed whether these factors enhanced specification of primitive streak cells to WT1 + nephron progenitors by Western blot. Unexpectedly, we observed the greatest WT1 expression in the absence of exogenous BMP7, RA, and FGF2 (Fig. 3B, C). Since we observed WT1 expression in the absence of exogenous BMPs, we then asked whether GSK3 inhibition induced endogenous BMP signaling. In the absence of dorsomorphin, which inhibits BMP type I receptors and blocks BMP-induced SMAD1/5/8 phosphorylation, the CHIR99021-treated cells exhibited high levels of phosphorylated SMAD1/5/8 (P-SMAD) at day 3 (Fig. 3D). However, when dorsomorphin was added from days 1–3 of differentiation, SMAD phosphorylation was completely inhibited at dorsomorphin concentrations greater than 5 µM. When dorsomorphin was added after removal of CHIR99021 (from day 3 to day 4), SMAD phosphorylation at day 4 was unaffected (Fig. 3E). We confirmed endogenous BMP7 production by immunofluorescence and Western blot of cell lysate at day 1 and day 2 of differentiation (Fig. 3F, G).

We then tested if dorsomorphin treatment inhibited nephron progenitor differentiation to assess whether endogenous BMP production plays a role in podocyte specification. Cells were analyzed for PAX2 and WT1 expression by flow cytometry at day 16 of differentiation. Cells treated with 1 µM dorsomorphin from day 1 to day 3 differentiated to WT1 and PAX2-expressing cells, although the expression levels of WT1 and PAX2 were significantly lower than in the untreated control (Fig. 3H, I). Together these results suggest that while GSK3 inhibition is sufficient to direct hPSCs to nephron progenitors, endogenous BMP7 signaling also plays a role.

### Nephron progenitors become mature podocytes

**Figure 4:**
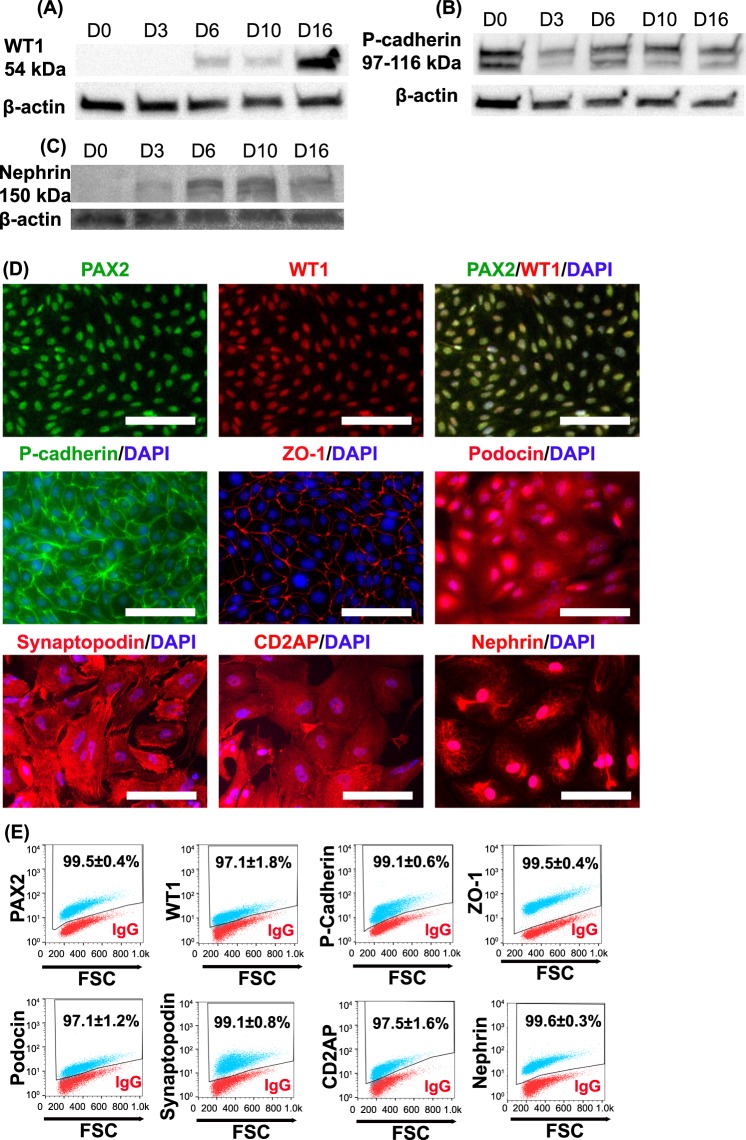
hPSC-derived podocytes express key podocyte markers. IMR90-4 iPSCs were differentiated as illustrated in Fig. 1A. Cell lysates were collected at days 0, 3, 6, 10, and 16 of differentiation. Western blot was used to assess the expression of (**A**) WT1, (**B**) P-cadherin, (**C**) nephrin. At day 16, cells differentiated as shown in Fig. 1A were characterized by immunofluorescence (**D**) and flow cytometry (**E**) for expression of the indicated podocyte proteins. Scale bars, 100 µm. In (**E**), red dots represent isotype control treated cells used to identify the gated regions and blue dots represent cells stained for the indicated marker. Numbers indicate the fraction of stained cells (blue) in the gated regions. Data are presented as mean ± SEM of three independent experiments. See also Figs S1–S5. Immunofluorescence labelling and flow cytometry were performed ten times from different differentiations on different days. Western blot was performed three times from three different differentiations.

Nephron progenitors differentiated as shown in Fig. 1A were characterized for acquisition of podocyte markers throughout the differentiation process by both qRT-PCR and Western blot. *CDH3* (P-cadherin), *SYNPO* (synaptopodin) and *TJP1* (ZO-1) expression gradually increased during the 16 day differentiation process (Fig. S1). WT1 protein was first detected at day 6 and was significantly more abundant at days 10 and 16 (Fig. 4A). P-cadherin is a cell-cell adhesion molecule that maintains the integrity of epithelial tissues^50^ and is expressed in hPSCs^51^. P-cadherin expression slightly decreased after iPSCs progressed to primitive streak and intermediate mesoderm at day 3 of differentiation (Fig. 4B). P-cadherin expression then increased and remained relatively constant from days 6–16. Both synaptopodin and nephrin are involved in the formation of the slit diaphragm^52,53^. During the differentiation process, significant nephrin expression was detected at day 6 and expression increased through day 16 (Fig. 4C). Immunofluorescence images were acquired at day 16 to show the localization of these key podocyte proteins (Fig. 4D). Nearly 100% of the day 16 IMR90–4 iPSC-derived podocytes expressed key podocyte proteins, including PAX2, WT1, P-cadherin, CD2AP, podocin, synaptopodin, nephrin and ZO-1, demonstrating the production of virtually pure podocytes from iPSCs (Fig. 4E). We also compared the expression of key podocyte proteins between hPSC-derived podocytes and human primary podocytes. Most major podocyte proteins, including PAX2, WT1, podocin, synaptopodin, ZO-1, CD2AP and nephrin were also expressed by primary podocytes. However, in the primary podocytes, the WT1 was not exclusively localized to the nucleus while P-cadherin expression was not detected, nor did we detect significant expression of WT1 or P-cadherin in primary podocytes by Western blot (Fig. S2). We used mouse kidney tissues as a positive control to validate the WT1 antibody for both Western blotting and immunofluorescence (Fig. S3A, B). We also verified that key markers were not expressed in undifferentiated hPSCs by immunofluorescence (Fig. S4A) and flow cytometry (Fig. S4B) for PAX2, WT1, MIXL1, SIX2, CD2AP, podocin, synaptopodin, and nephrin. MIXL1 was also not detected in day 16 cells by flow cytometry (Fig. S4B). While PAX2 is downregulated in mature podocytes *in vivo*^54^, hPSC-derived podocytes and primary podocytes have been reported to reacquire or maintain PAX2 expression^34,55^.

We then tested the differentiation protocol illustrated in Fig. 1A in two additional hPSC lines. At day 16, nearly 100% of the cells differentiated from both H9 hESCs and 19-9-11 iPSCs expressed key podocyte proteins, including PAX2, WT1, P-cadherin, ZO-1, CD2AP, podocin, synaptopodin, nephrin (Fig. S5). To further define the differentiation system, we also tested podocyte differentiation on defined substrates, Synthemax and vitronectin, as replacements for Matrigel. Almost 100% of the cells differentiated on both Synthemax- and vitronectin-coated surfaces showed high expression of podocyte proteins, similar to differentiation on Matrigel (Fig. S6). For IMR90-4 iPSC line, we performed the optimized differentiation process more than 30 times. For both H9 ESCs and 19-9-11 iPSCs, we performed the experiments at least three independent times. For differentiation on Synthemax- and vitronectin-coated surfaces, we performed experiments at least three times.

### hPSC-derived podocytes exhibit podocyte phenotypes

**Figure 5:**
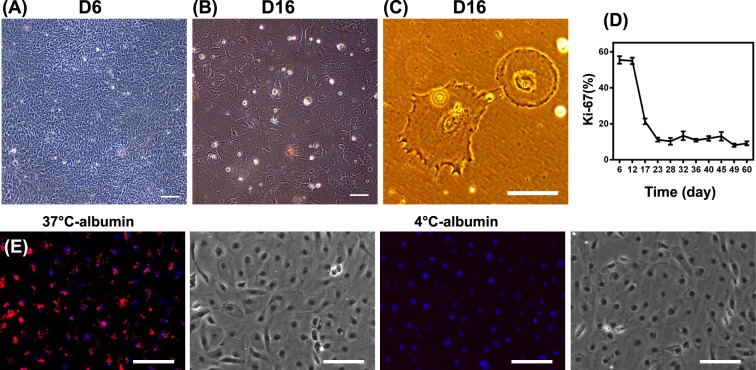
hPSC-derived podocytes exhibit key podocyte phenotypes. IMR90-4 iPSC-derived podocytes were differentiated as illustrated in Fig. 1A. Phase contrast images of IMR90-4 iPSC-derived podocytes were taken at (**A**) day 6, and (**B**,**C**) day 16. (**D**) Proliferation of cells at different time points was assessed by flow cytometry for Ki67. (**E**) IMR90-4 iPSC-derived podocytes at day 16 were analyzed with an Albumin Uptake Assay Kit. Alexa Fluor™ 555-labelled albumin is shown in red on a merged DAPI image and the corresponding bright field image is provided on the right. 4 °C was used as a control to prevent endocytosis. Scale bars, 100 µm. Albumin uptake assay was performed three times from three different differentiations.

Given the marker expression profile characteristic of podocytes, we examined several podocyte phenotypes at day 16. Nephron progenitors are proliferative and primary podocytes exhibit a cobblestone-like morphology in primary cell culture^56^. IMR90-4 iPSC-derived nephron progenitors exhibited a cobblestone-like shape at day 6 of differentiation (Fig. 5A), very similar to the morphology of primary human podocytes under standard culture condition (Fig. S6A). After 16 days, these iPSC-derived podocytes formed a monolayer of large arborized cells exhibiting prominent thin processes (Fig. 5B, C), consistent with the morphology of fully differentiated podocytes^19^. Differentiated primary podocytes lack the capacity to divide in culture^56^. Similarly, IMR90-4 iPSC-derived podocytes lost proliferative capacity during differentiation, as determined by Ki-67 staining, after day 12 of the differentiation. By day 22 only about 10% of cells expressed Ki-67 (Fig. 5D). At day 16, 70 ± 10 podocytes were produced per undifferentiated iPSC at day 0, indicating substantial expansion during the differentiation process. Thus, by day 16 of differentiation, the iPSCs have progressed through a nephron progenitor stage to non-proliferative cells expressing hallmark podocyte markers.

**Figure 6:**
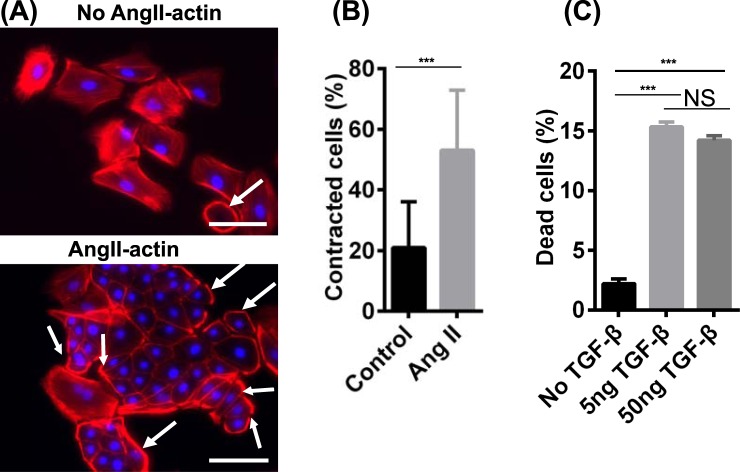
hPSC-derived podocytes exhibit actin reorganization after angiotensin II treatment and cell death is induced by TGF-β1 treatment. IMR90-4 iPSC-derived podocytes were differentiated as illustrated in Fig. 1A. (**A**) At day 16, cells were treated with 500 ng/mL Ang II for 6 hr and phalloidin staining was used to assess changes in cytoskeletal structure. White arrows indicate the cells with peripheral F-actin. (**B**) More than 200 cells over 15 images were analyzed and the percentage of cells with peripherally-organized actin was calculated for control and Ang II-treated cells. Data are presented as mean percentage ± SEM of three independent experiments over 15 images. Scale bars, 100 µm. (**C**) At day 16, cells were treated with 5 ng/mL and 50 ng/mL TGF-β1for 24 hr. Detached cells were collected from the medium and combined with Accutase-dissociated cells from the substrate. Cells were treated with trypan blue and the numbers of cells incorporating and excluding trypan blue were counted on a hemocytometer. Percentage of dead cells was calculated as the number of cells that incorporated trypan blue divided by the total cell number. Data are presented as mean ± SEM of three independent experiments. Ang II and TGF-β treat experiments were performed three times from three different differentiations.

Podocytes uptake albumin via endocytosis and degrade albumin in lysosomes^57,58^. We assessed the ability of day 16 iPSC-derived podocytes to endocytose Alexa Fluor 555-labeled albumin in a temperature-dependent manner. At 37 °C, the iPSC-derived podocytes contained extensive fluorescence in intercellular vesicles, while the cells at 4 °C, where endocytosis is inhibited, did not incorporate albumin (Fig. 5E). We further confirmed temperature-dependent albumin uptake in human primary podocytes (Fig. S7B). Next, angiotensin II (Ang II) induces podocyte damage and glomerular disease in part by altering cytoskeletal structure and inducing contractility^59^. To examine the effects of Ang II, we treated day 16 iPSC-derived podocytes with 500 ng/mL Ang II for 6 hr. Ang II-treated cells displayed a reorganized actin morphology compared to untreated cells (Fig. 6A, indicated with white arrows). Over 50% of the IMR90-4 iPSC-derived podocytes exhibited peripheral actin upon Ang II stimulation, while only about 20% of control cells exhibited peripheral actin (Fig. 6B). Finally, TGF-β1 induces cell death in primary and immortalized podocytes^60,61^. Hence, we tested the effects of TGF-β1 treatment on day 16 IMR90-4 iPSC-derived podocyte viability. iPSC-derived podocytes exposed to 5 or 50 ng/mL TGF-β1 for 24 hr exhibited a significantly higher percentage of dead cells than the control (15% vs. 2%) as assessed by trypan blue uptake (Fig. 6C). Taken together, iPSC-derived podocytes display phenotypes that have been described for terminally differentiated podocytes.

## Discussion

In this study, we demonstrate a simple method to differentiate hPSCs into terminally-differentiated podocytes in a defined system. After treatment with the small molecule GSK3ß inhibitor CHIR99021, hPSCs differentiate in a developmentally-relevant progression from the pluripotent stage through primitive streak-like cells, nephron progenitors and eventually to mature podocytes that express key podocyte proteins, including PAX2, WT1, podocin, synaptopodin and nephrin. Fully differentiated podocytes do not proliferate significantly *in vivo* or *in vitro*^62^. Likewise, hPSC-derived podocytes lost proliferative capacity after day 16 of differentiation. Importantly, hPSC-derived podocytes exhibit key podocyte phenotypes, including actin reorganization upon Ang II stimulation, albumin uptake and induction of cell death upon TGF-β1 treatment. This differentiation protocol employs a defined system, including serum-free culture medium as well as a defined extracellular matrix. Defined systems generally enhance reproducibility and facilitate production of cells for therapeutic applications^63,64^.

*In vivo*, the majority cell types forming the kidney, including podocytes, originate from intermediate mesoderm^36^. Canonical Wnt signaling activation has been shown to play an important role in the differentiation of hPSCs into intermediate mesoderm^37–39^. Previously, several studies have shown that Wnt pathway activation combined with BMP4, BMP7, RA and FGF2 treatment is essential to differentiate hPSCs into podocytes^32–34^. We determined that application of 6 µM CHIR99021 alone, in a podocyte permissive medium, was sufficient to direct three different hPSC lines into intermediate mesoderm that then became nephron progenitors and eventually virtually pure populations of mature podocytes as assessed by flow cytometry for podocyte markers. We also found that BMP7, RA or FGF2 treatment actually diminished WT1 expression in the hPSC-derived podocytes. Moreover, in the differentiation process described here, BMP7 was endogenously expressed and SMAD1/5/8 activated in the differentiating hPSCs. Treatment with the BMP inhibitor dorsomorphin dramatically decreased podocyte differentiation efficiency, suggesting this endogenous BMP signaling is necessary for podocyte specification. This finding is consistent with a prior report that canonical Wnt signaling activates the BMP pathway in skeletal myoblasts by inducing BMP4 expression^65^. While it is not clear why BMP7 addition reduced WT1 expression, autocrine or paracrine BMP signaling appears to be necessary for podocyte specification in our differentiation process, perhaps induced as a consequence of Wnt pathway activation^66^, obviating the need for exogenous BMP ligands, as required by all other reported podocyte differentiation protocols. Compared to previous podocyte differentiation protocols^32–34^, although all these methods are very straightforward and simple, the differentiation process described here does not require BMP7, FGF2, and RA. With fewer growth factors or small molecules in the differentiation, it is potentially simpler to troubleshoot and adapt to different applications, including generating clinical-grade podocytes for disease modeling and drug screening.

Compared to human primary podocytes, which are nonproliferative *in vitro*, hPSCs can be used to generate large quantities of healthy or patient-specific human podocytes for disease modeling, drug screening, and development of cell-based therapies. Podocytes have limited capacity for repair or regeneration; thus, podoycte loss is a central feature of many forms of progressive CKD. hPSC-derived podocytes represent an attractive modality to study podocyte injury compared to primary cultures of podocytes which lack proliferation capacity *in vitro*. In this way, hPSC-derived podocytes could be used to study human glomerular diseases and for screening effects of drugs on glomeruli *in vivo*. hPSC-derived podocytes also have been shown to reconstitute kidney glomerular-capillary-wall function on a microfluidic chip and this platform may have personalized-medicine applications for diseases due to inherited deficiencies of podocyte genes^33^. hPSC-derived podocytes have been transplanted into mouse kidneys and integrated with glomeruli^34,67^, indicating their potential importance in transplantation and kidney regeneration applications. The podocyte differentiation approach reported here utilizes fully defined system and allows the robust generation of podocytes, which can potentially be used for such modeling and cell therapy applications.

## Methods

### hPSC culture and differentiation

hPSCs (iPS(IMR90)-4^68^, iPS-DF 19-9-11T^69^, and hESCs (H9)^70^) were maintained on Matrigel (Corning)-coated surfaces in mTeSR1 (STEMCELL Technologies) as previously described^71^. Before differentiation, hPSCs were singularized with Accutase (Innovative Cell Technologies) and plated onto Matrigel-coated (or 25 µg/mL Synthemax, or 5 µg/mL vitronectin Thermo Fisher) plates at a density of ~2 × 10^4^ cells/cm^2^ in mTeSR1 supplemented with 10 μM ROCK inhibitor Y-27632 (Selleckchem). hPSCs were expanded in mTeSR1 for three days. At day 0, differentiation was initiated by treating cells with 6 μM CHIR99021 (Selleckchem) in podocyte medium 1 (PM1): DMEM/Ham’s F12 (Thermo Fisher), 1% MEM nonessential amino acids (Thermo Fisher), 0.5% GlutaMAX (Thermo Fisher), and 0.1 mM β-mercaptoethanol (SigmaAldrich) for 2 days. After 48 hr, medium was changed to podocyte medium 2 (PM2): human Endothelial Serum-Free Medium (hESFM) (Thermo Fisher) supplemented with 2% B27. After 4 days of culture in PM2, day 6 cells were dissociated with Accutase and plated at a 1:6 ratio (approximately 4 × 10^4^ cells/cm^2^) in PM2 onto 12-well tissue culture plates coated with 100 μg/mL Matrigel (or 25 µg/mL Synthemax, or 5 µg/mL vitronectin). At day 10, after reaching confluence, cells were split again at a ratio of 1:3. Cells were cultured in PM2 after differentiation day 2 and medium was changed every day for the first 10 days. After day 10, medium was replaced every other day. For differentiation of iPSC IMR90-4, 23 different differentiations on different days were performed. For iPSC 19-9-11 and hESC H9 differentiation, three differentiations were performed on different days. For differentiation on vitronectin and Synthemax, three differentiations were performed on different days.

### Primary human podocyte culture

Human primary podocytes were purchased from Celprogen and cultured on human podocyte primary cell culture Extra-cellular Matrix 12 Well Plates (Celprogen) supplied with human podocyte primary cell culture complete media with Serum (Celprogen).

### Immunochemistry

Cells were rinsed with ice-cold PBS once and fixed with 4% paraformaldehyde (PFA, Electron Microscopy Sciences) for 15 min. Cells were then blocked with 10% goat serum (Thermo Fisher) in PBS (SigmaAldrich) containing 0.3% Triton-X100 (Fisher Scientific) for 30 min (10% PBSGT). Primary antibodies were diluted in 10% PBSGT and cells were incubated in the antibody solutions at 4 °C overnight or at room temperature for 2 hr. After three PBS washes, cells were incubated with secondary antibodies in 10% PBGST (goat anti-rabbit Alexa Fluor 594 (Invitrogen) and goat anti-mouse Alexa Fluor 488 (Invitrogen); 1:200) for 1 hr at room temperature. Cells were then washed with PBS three times followed by treatment with DAPI fluoromount-G (Southern Biotech) and visualized. A list of antibody sources and dilutions is provided in Table S1.

### Western blot assay

Cells were dissociated with Accutase and rinsed with PBS twice before being lysed with RIPA (Rockland) in the presence of 1% of Halt Protease and Phosphatase Inhibitor Cocktail (Pierce). Protein concentration was determined by a BCA assay kit (Thermo Fisher) according to manufacturer’s instructions. Samples containing 30 µg of total protein were loaded onto pre-cast 10% Tris-Glycine SDS/PAGE gels (Invitrogen) under denaturing conditions and transferred to a nitrocellulose membrane. After blocking with 5% non-fat milk in TBST, the membrane was incubated with primary antibody (Table S1) overnight at 4 °C. The membrane was then washed, incubated with an anti-mouse/rabbit peroxidase-conjugated secondary antibody (Table S1) for 1 hr at room temperature or overnight at 4 °C, and developed by SuperSignal chemiluminescence (Pierce).

### Flow cytometry

Cells were dissociated with Accutase and fixed in 1% PFA for 15 min at room temperature, then washed with 0.5% BSA (Bio-Rad) plus 0.1% Triton-X100 three times. Cells were stained with primary and secondary antibodies diluted in 0.5% BSA plus 0.1% Triton-X 100 as described^37^. Data were collected on a FACS Caliber flow cytometer (Beckton Dickinson) and analyzed using FlowJo. Corresponding isotype antibodies were used as FACS gating control. Details about antibody source and usage are provided in Table S1.

### Quantitative RT-PCR

Total RNA was extracted with the RNeasy mini kit (QIAGEN) and treated with DNase (QIAGEN). 1 μg total RNA was reverse transcribed into cDNA using Oligo (dT) primer with Superscript III Reverse Transcriptase (Invitrogen). Real-time quantitative PCR was done in triplicate with iQSYBR Green SuperMix (Bio-Rad). *GAPDH* was used as an endogenous housekeeping control. Primer sequences are provided in Table S2.

### Albumin uptake assay

Differentiated podocytes at day 16 were incubated with the Alexa Fluor™ 555 conjugated albumin (Thermo Fisher) at 500 µg/mL for 1 hr at 37 °C. Cells incubated with same concentration of albumin at 4 °C were used as a control. The cells were then rinsed with ice-cold PBS three times and fixed with 4% PFA. Nuclei were stained with DAPI and Alexa Fluor^TM^ 555 conjugated albumin was visualized.

### Contractility assay and cell death assay

Differentiated podocytes at day 16 were treated with 500 ng/mL Ang II for 6 hr or 5 ng/mL (or 50 ng/mL) TGF-β1 for 24 hr. After 6 hr, the cells treated with Ang II were fixed with 4% PFA and stained with DyLight-conjugated phalloidin (Thermo Fisher). Nuclei were stained with DAPI and phalloidin was visualized. The cells treated with TGF-β1 after 24 hr were assessed by counting cells that excluded trypan blue.

### Statistics

Data are presented as mean ± standard error of the mean (SEM). Statistical significance was determined by Student’s t-test (two-tail) between two groups. P < 0.05 was considered statistically different.

## Supplementary information

Supplementary Information

## References

[1] Pavenstädt H, Kriz W, Kretzler M (2003). Cell biology of the glomerular podocyte. Physiological reviews.

[2] Ryan GB, Karnovsky MJ (1976). Distribution of endogenous albumin in the rat glomerulus: role of hemodynamic factors in glomerular barrier function. Kidney international.

[3] Haraldsson B, Nyström J, Deen WM (2008). Properties of the glomerular barrier and mechanisms of proteinuria. Physiological reviews.

[4] Schwarz K (2001). Podocin, a raft-associated component of the glomerular slit diaphragm, interacts with CD2AP and nephrin. The Journal of clinical investigation.

[5] Nangaku M (2006). Chronic hypoxia and tubulointerstitial injury: a final common pathway to end-stage renal failure. Journal of the American Society of Nephrology.

[6] Boute N (2000). NPHS2, encoding the glomerular protein podocin, is mutated in autosomal recessive steroid-resistant nephrotic syndrome. Nature genetics.

[7] Foley RN, Parfrey PS, Sarnak MJ (1998). Clinical epidemiology of cardiovascular disease in chronic renal disease. American Journal of Kidney Diseases.

[8] Koffler D, Schur PH, Kunkel HG (1967). Immunological studies concerning the nephritis of systemic lupus erythematosus. Journal of Experimental Medicine.

[9] Wilson CB, Smith RC (1972). Goodpasture’s syndrome associated with influenza A2 virus infection. Ann Intern Med.

[10] Radford MG, Donadio JV, Bergstralh EJ, Grande JP (1997). Predicting renal outcome in IgA nephropathy. Journal of the American Society of Nephrology.

[11] Neugarten J, Baldwin DS (1984). Glomerulonephritis in bacterial endocarditis. The American journal of medicine.

[12] Barisoni L, Kriz W, Mundel P, D’AGATI V (1999). The Dysregulated Podocyte Phenotype A Novel Concept in the Pathogenesis of Collapsing Idiopathic Focal Segmental Glomerulosclerosis and HIV-Associated Nephropathy. Journal of the American Society of Nephrology.

[13] Kashtan CE (1998). Alport syndrome and thin glomerular basement membrane disease. Journal of the American Society of Nephrology.

[14] Sassy-Prigent C (2000). Early glomerular macrophage recruitment in streptozotocin-induced diabetic rats. Diabetes.

[15] Baylis C, Mitruka B, Deng A (1992). Chronic blockade of nitric oxide synthesis in the rat produces systemic hypertension and glomerular damage. Journal of Clinical Investigation.

[16] Wiggins R-C (2007). The spectrum of podocytopathies: a unifying view of glomerular diseases. Kidney international.

[17] Saran R (2017). US renal data system 2016 annual data report: epidemiology of kidney disease in the United States. American journal of kidney diseases.

[18] Couser WG, Remuzzi G, Mendis S, Tonelli M (2011). The contribution of chronic kidney disease to the global burden of major noncommunicable diseases. Kidney international.

[19] Saleem MA (2002). A conditionally immortalized human podocyte cell line demonstrating nephrin and podocin expression. Journal of the American Society of Nephrology.

[20] Susztak K, Raff AC, Schiffer M, Böttinger EP (2006). Glucose-induced reactive oxygen species cause apoptosis of podocytes and podocyte depletion at the onset of diabetic nephropathy. Diabetes.

[21] Durvasula RV (2004). Activation of a local tissue angiotensin system in podocytes by mechanical strain. Kidney international.

[22] Pavenstädt H (2000). Roles of the podocyte in glomerular function. American Journal of Physiology-Renal Physiology.

[23] Wharram BL (2005). Podocyte depletion causes glomerulosclerosis: Diphtheria toxin–induced podocyte depletion in rats expressing human diphtheria toxin receptor transgene. Journal of the American Society of Nephrology.

[24] Mundel P (1997). Rearrangements of the cytoskeleton and cell contacts induce process formation during differentiation of conditionally immortalized mouse podocyte cell lines. Experimental cell research.

[25] Chittiprol S, Chen P, Petrovic-Djergovic D, Eichler T, Ransom RF (2011). Marker expression, behaviors, and responses vary in different lines of conditionally immortalized cultured podocytes. American Journal of Physiology-Renal Physiology.

[26] Takasato M (2014). Directing human embryonic stem cell differentiation towards a renal lineage generates a self-organizing kidney. Nature cell biology.

[27] Xia Y (2013). Directed differentiation of human pluripotent cells to ureteric bud kidney progenitor-like cells. Nature cell biology.

[28] Takasato M (2015). Kidney organoids from human iPS cells contain multiple lineages and model human nephrogenesis. Nature.

[29] Taguchi A, Nishinakamura R (2017). Higher-Order Kidney Organogenesis from Pluripotent Stem Cells. Cell stem cell.

[30] Morizane R (2015). Nephron organoids derived from human pluripotent stem cells model kidney development and injury. Nature biotechnology.

[31] Taguchi A (2014). Redefining the *in vivo* origin of metanephric nephron progenitors enables generation of complex kidney structures from pluripotent stem cells. Cell stem cell.

[32] Ciampi O (2016). Generation of functional podocytes from human induced pluripotent stem cells. Stem cell research.

[33] Musah S (2017). Mature induced-pluripotent-stem-cell-derived human podocytes reconstitute kidney glomerular-capillary-wall function on a chip. Nature Biomedical Engineering.

[34] Song B (2012). The directed differentiation of human iPS cells into kidney podocytes. PloS one.

[35] Kang M, Han Y-M (2014). Differentiation of human pluripotent stem cells into nephron progenitor cells in a serum and feeder free system. PloS one.

[36] Mae SI (2013). Monitoring and robust induction of nephrogenic intermediate mesoderm from human pluripotent stem cells. Nat Commun.

[37] Lian X (2012). Robust cardiomyocyte differentiation from human pluripotent stem cells via temporal modulation of canonical Wnt signaling. Proceedings of the National Academy of Sciences.

[38] Marcelle C, Stark MR, Bronner-Fraser M (1997). Coordinate actions of BMPs, Wnts, Shh and noggin mediate patterning of the dorsal somite. Development.

[39] Perantoni, A. O. In Seminars in cell & developmental biology. 201-208 (Elsevier).

[40] Lian X (2015). Chemically defined, albumin-free human cardiomyocyte generation. Nature methods.

[41] Qian T (2017). Directed differentiation of human pluripotent stem cells to blood-brain barrier endothelial cells. Science Advances.

[42] Kreidberg JA (2010). WT1 and kidney progenitor cells. Organogenesis.

[43] Davis AP, Witte DP, Hsieh-Li HM, Potter SS, Capecchi MR (1995). Absence of radius and ulna in mice lacking hoxa-11 andhoxd-11. Nature.

[44] Patterson LT, Pembaur M, Potter SS (2001). Hoxa11 and Hoxd11 regulate branching morphogenesis of the ureteric bud in the developing kidney. Development.

[45] Obara-Ishihara T, Kuhlman J, Niswander L, Herzlinger D (1999). The surface ectoderm is essential for nephric duct formation in intermediate mesoderm. Development.

[46] Xia Y (2014). The generation of kidney organoids by differentiation of human pluripotent cells to ureteric bud progenitor–like cells. Nature protocols.

[47] Mendelsohn C (1994). Function of the retinoic acid receptors (RARs) during development (II). Multiple abnormalities at various stages of organogenesis in RAR double mutants. Development.

[48] Duester G (2008). Retinoic acid synthesis and signaling during early organogenesis. Cell.

[49] Burrow CR (2000). Regulatory molecules in kidney development. Pediatric nephrology.

[50] Vieira AF, Paredes J (2015). P-cadherin and the journey to cancer metastasis. Molecular cancer.

[51] Chen H-F (2011). Surface marker epithelial cell adhesion molecule and E-cadherin facilitate the identification and selection of induced pluripotent stem cells. Stem Cell Reviews and Reports.

[52] Yanagida-Asanuma E (2007). Synaptopodin protects against proteinuria by disrupting Cdc42: IRSp53: Mena signaling complexes in kidney podocytes. The American journal of pathology.

[53] Ruotsalainen V (1999). Nephrin is specifically located at the slit diaphragm of glomerular podocytes. Proceedings of the National Academy of Sciences.

[54] Lasagni L (2015). Podocyte regeneration driven by renal progenitors determines glomerular disease remission and can be pharmacologically enhanced. Stem cell reports.

[55] Kabgani N (2012). Primary cultures of glomerular parietal epithelial cells or podocytes with proven origin. PloS one.

[56] Shankland S, Pippin J, Reiser J, Mundel P (2007). Podocytes in culture: past, present. and future. Kidney international.

[57] Xinaris, C. *et al*. Functional human podocytes generated in organoids from amniotic fluid stem cells. *Journal of the American Society of Nephrology, ASN*. 2015030316 (2015). 10.1681/ASN.2015030316 PMC4849826 26516208

[58] Carson JM (2014). Podocytes degrade endocytosed albumin primarily in lysosomes. PLoS One.

[59] Kriz W (1994). A role for podocytes to counteract capillary wall distension. Kidney international.

[60] Schiffer M (2001). Apoptosis in podocytes induced by TGF-β and Smad7. The Journal of clinical investigation.

[61] Das R (2015). Transforming growth factor β1-induced apoptosis in podocytes via the extracellular signal-regulated kinase-mammalian target of rapamycin complex 1-NADPH oxidase 4 axis. Journal of Biological Chemistry.

[62] Griffin SV, Petermann AT, Durvasula RV, Shankland SJ (2003). Podocyte proliferation and differentiation in glomerular disease: role of cell-cycle regulatory proteins. Nephrology Dialysis Transplantation.

[63] Kirouac DC, Zandstra PW (2008). The systematic production of cells for cell therapies. Cell stem cell.

[64] Serra M, Brito C, Correia C, Alves PM (2012). Process engineering of human pluripotent stem cells for clinical application. Trends in biotechnology.

[65] Kuroda K, Kuang S, Taketo MM, Rudnicki MA (2013). Canonical Wnt signaling induces BMP-4 to specify slow myofibrogenesis of fetal myoblasts. Skeletal muscle.

[66] Cho YD (2012). Wnt3a stimulates Mepe, Matrix extracellular phosphoglycoprotein, expression directly by the activation of the canonical Wnt signaling pathway and indirectly through the stimulation of autocrine Bmp‐2 expression. Journal of cellular physiology.

[67] Sharmin, S. *et al*. Human induced pluripotent stem cell–derived podocytes mature into vascularized glomeruli upon experimental transplantation. *Journal of the American Society of Nephrology, ASN*. 2015010096 (2015). 10.1681/ASN.2015010096 PMC4884101 26586691

[68] Yu J (2007). Induced pluripotent stem cell lines derived from human somatic cells. Science.

[69] Yu J (2009). Human induced pluripotent stem cells free of vector and transgene sequences. Science.

[70] Thomson JA (1998). Embryonic stem cell lines derived from human blastocysts. science.

[71] Ludwig TE (2006). Feeder-independent culture of human embryonic stem cells. Nature methods.

