# Supplementary material for: Directed Differentiation of Human Pluripotent Stem Cells to Podocytes under Defined Conditions
Source: Sci Rep. 2019 Feb 26;9:2765. doi: 10.1038/s41598-019-39504-8 (PMC6391455; doi:10.1038/s41598-019-39504-8)
Supplement: Supplementary file 1 — Supplementary Information [file 41598_2019_39504_MOESM1_ESM.pdf]

Directed Differentiation of Human Pluripotent Stem Cells to Podocytes under Defined Conditions

Tongcheng Qian[1], Shaenah E. Hernday[1], Xiaoping Bao[1], William R. Olson[1], Sarah E. Panzer[2], Eric V. Shusta[1*] & Sean P. Palecek[1*]

[1]Department of Chemical & Biological Engineering, University of Wisconsin, Madison, WI, 53706, USA.
[2]School of Medicine and Public Health, University of Wisconsin, Madison, WI, 53706, USA.

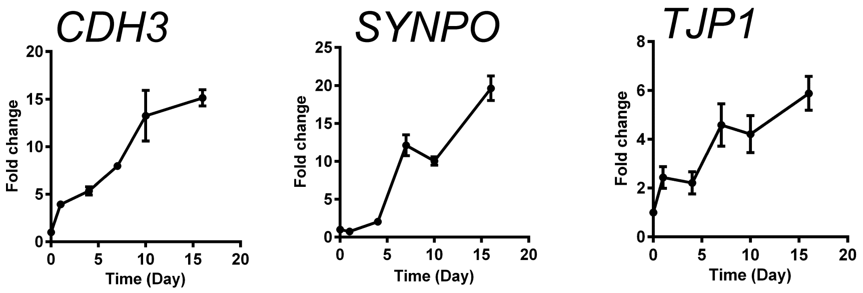

Figure S1. Gene expression during podocyte differentiation. Related to Figure 4. Quantitative RT-PCR was used to quantify the expression of the podocyte markers *CDH3* (P-cadherin), *SYNPO* (synaptopodin) and *TJP1* (ZO-1) during IMR90-4 iPSC differentiation to podocytes using the protocol illustrated in Fig. 1A. *GAPDH* was used as an endogenous housekeeping control and expression levels were normalized to day 0. Data are presented as mean ± SEM of three independent experiments.

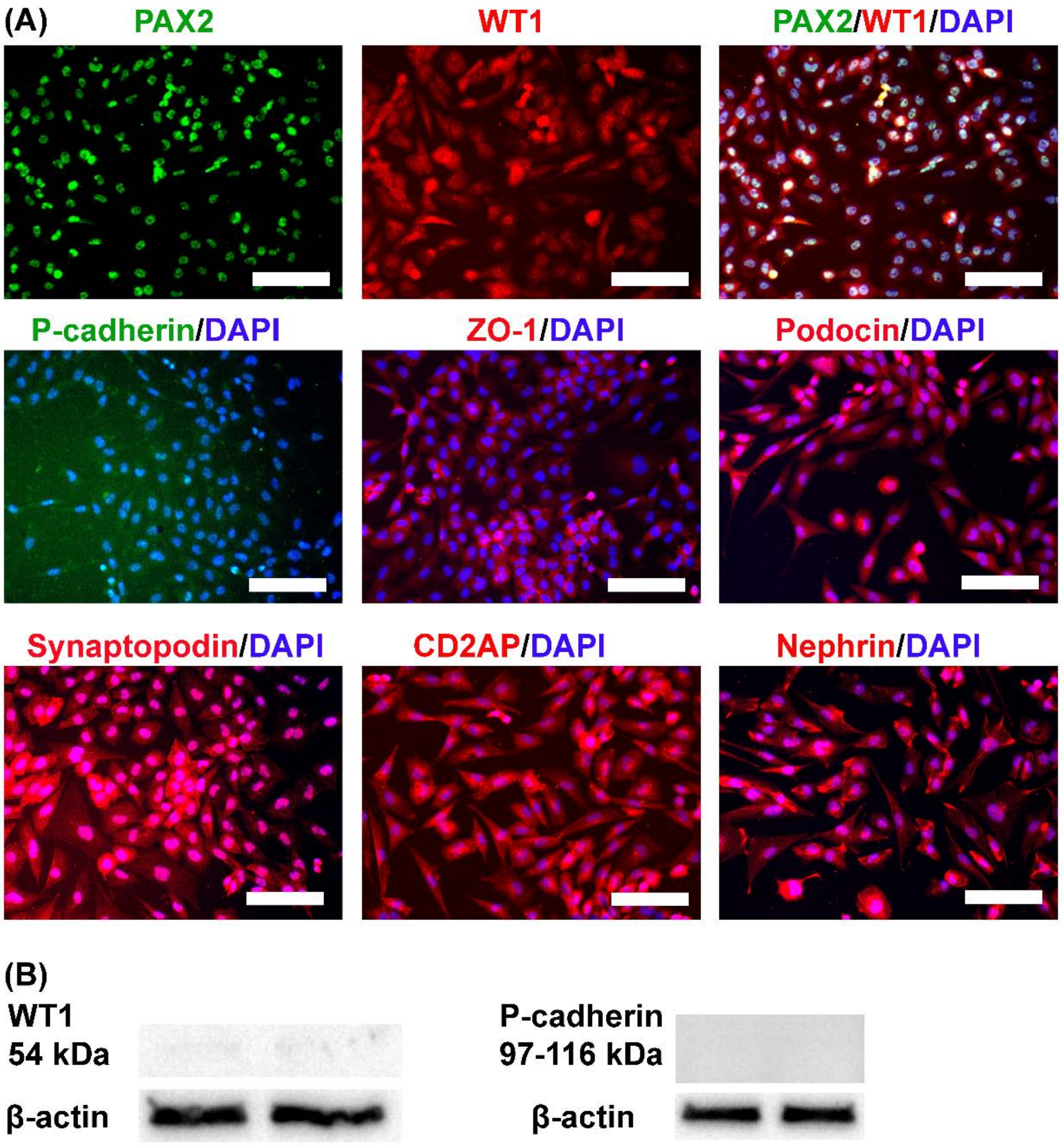

Figure S2. Primary podocytes express most podocyte proteins. (A) Primary podocytes were characterized by immunofluorescence for the indicated proteins. (B) Western blot was used to assess the expression of WT1 and P-cadherin in primary podocytes. Scale bars, 100 µm.

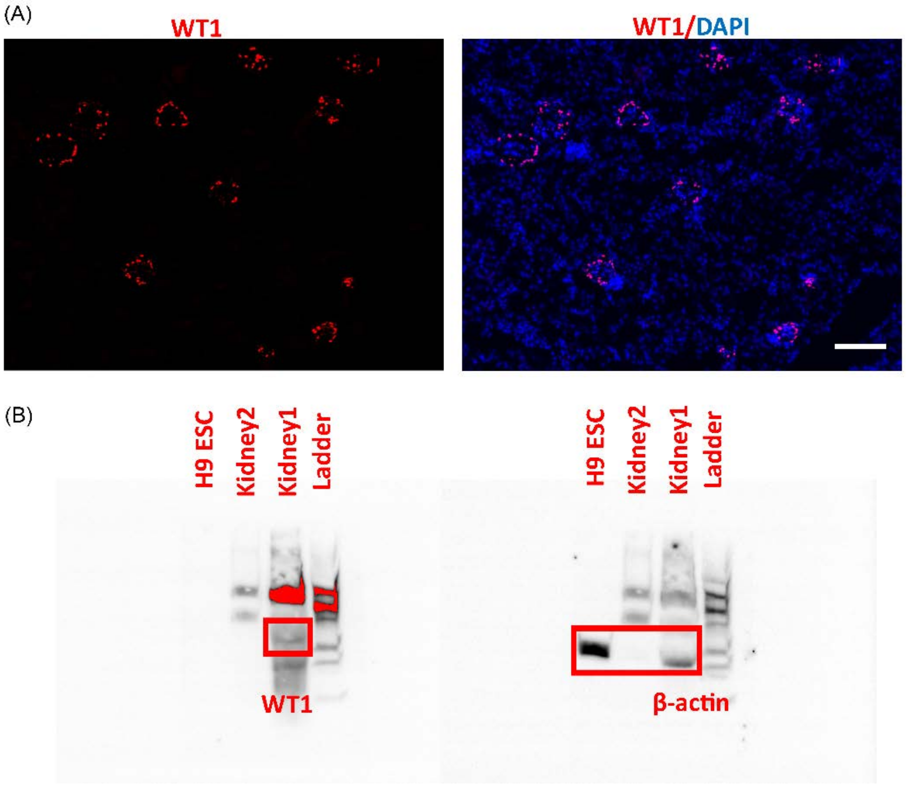

Figure S3. WT1 is expressed in mouse kidney tissues but not undifferentiated hPSCs or hPSCs after 24 hr CHIR treatment . (A) An 8 µm mouse kidney tissue section was labelled with WT1 (red) by immunofluorescence and merged with DAPI staining (blue). (B) WT1 expression in H9 hESCs, Kidney1 (lysed mouse kidney tissue), Kidney2 (Kidney1 samples lysed a second time) was analyzed by Western blot. Boxed bands indicate bands at the expected size. Scale bar, 100 µm.

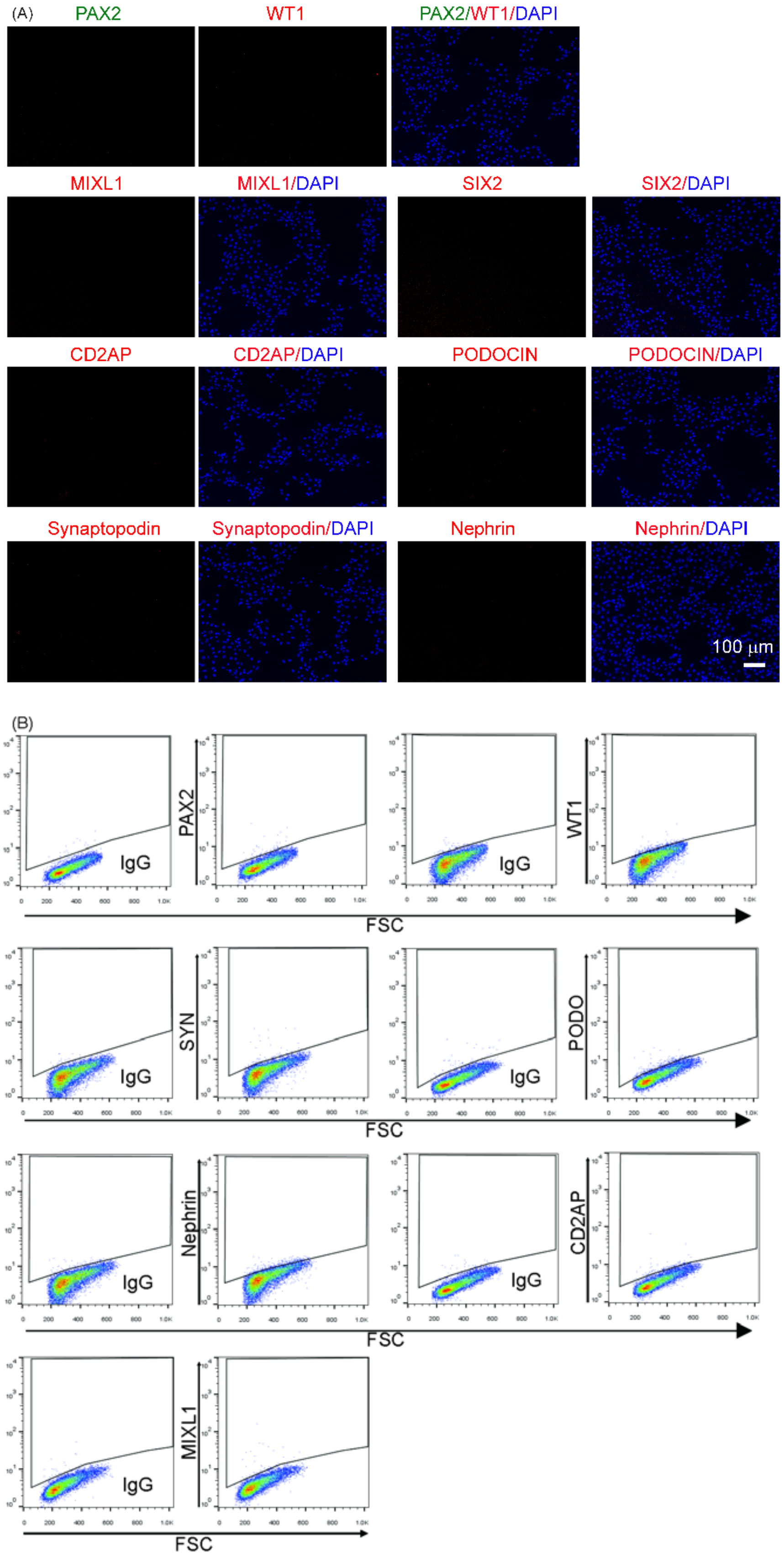
(A)
PAX2
WT1
PAX2/WT1/DAPI
MIXL1
MIXL1/DAPI
SIX2
SIX2/DAPI
CD2AP
CD2AP/DAPI
PODOCIN
PODOCIN/DAPI
Synaptopodin
Synaptopodin/DAPI
Nephrin
Nephrin/DAPI
100 μm
(B)
PAX2
WT1
IgG
FSC
SYN
PODO
Nephrin
CD2AP
MIXL1

Figure S4. PAX2, WT1, MIXL1, SIX2, CD2AP, Podocin, Synaptopodin, and Nephrin were not detected in undifferentiated IMR90-4 iPSCs. (A) Undifferentiated iPSCs were immunostained for the indicated marker and stained with DAPI then imaged. (B) PAX2, WT1, SIX2, CD2AP, Podocin, Synaptopodin, and nephrin were not detected in undifferentiated IMR90-4 iPSCs by flow cytometry (B). MIXL1 was not detected by flow cytometry in day 16 cells. IgG represents signal from isotype control antibodies. Scale bar, 100 µm.

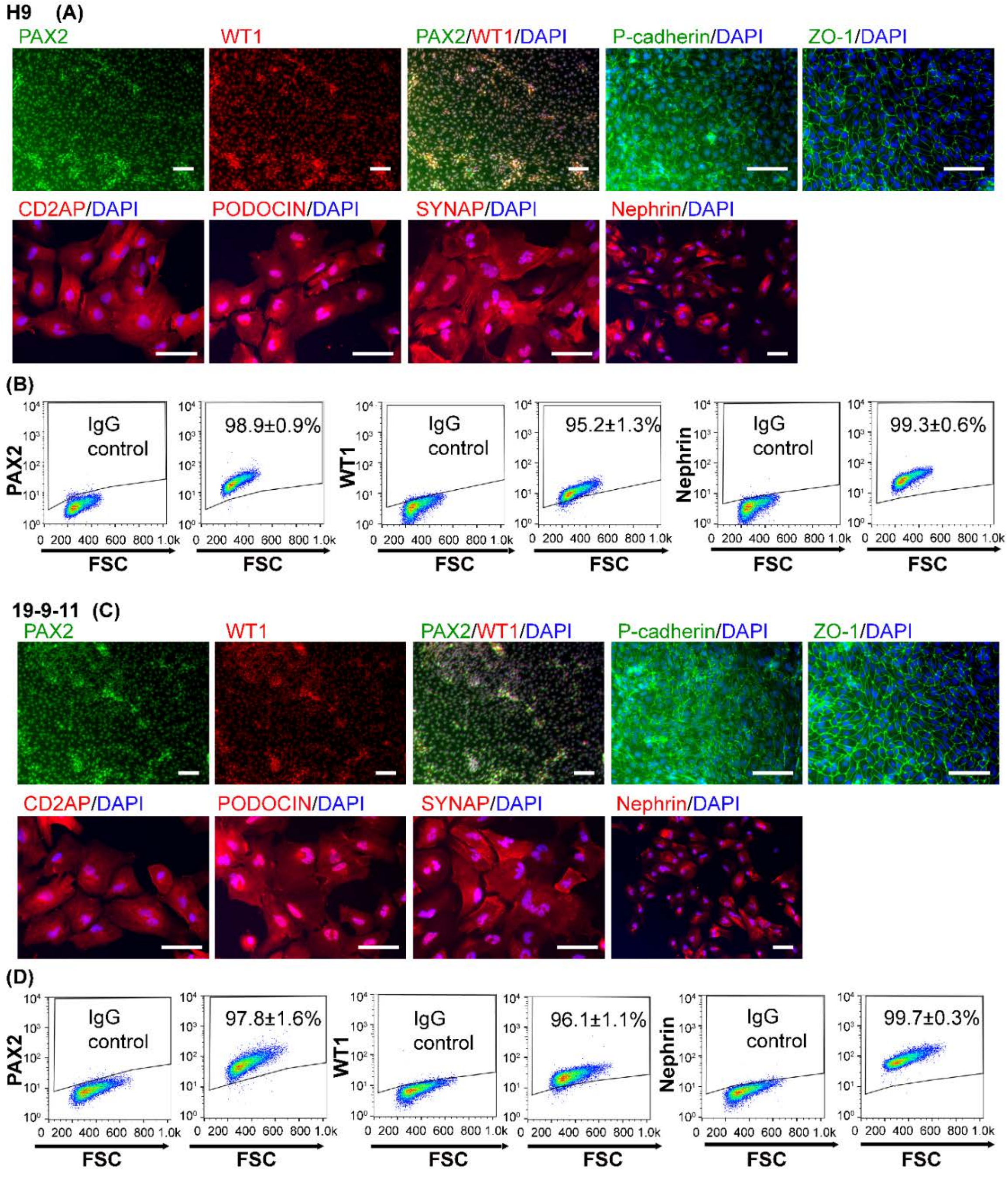

Figure S5. Podocytes differentiated from H9 hESCs and 19-9-11 iPSCs express podocyte-related proteins. Related to Figure 4. Podocytes were differentiated as illustrated in Fig. 1A. At day 16, expression of the indicated proteins was assessed by immunofluorescence (A, C) and flow cytometry (B, D). The numbers on the flow cytometry plots indicate the percentage of cells in the gated region. Data are presented as mean ± SEM of three independent experiments. Scale bars, 100 μm.

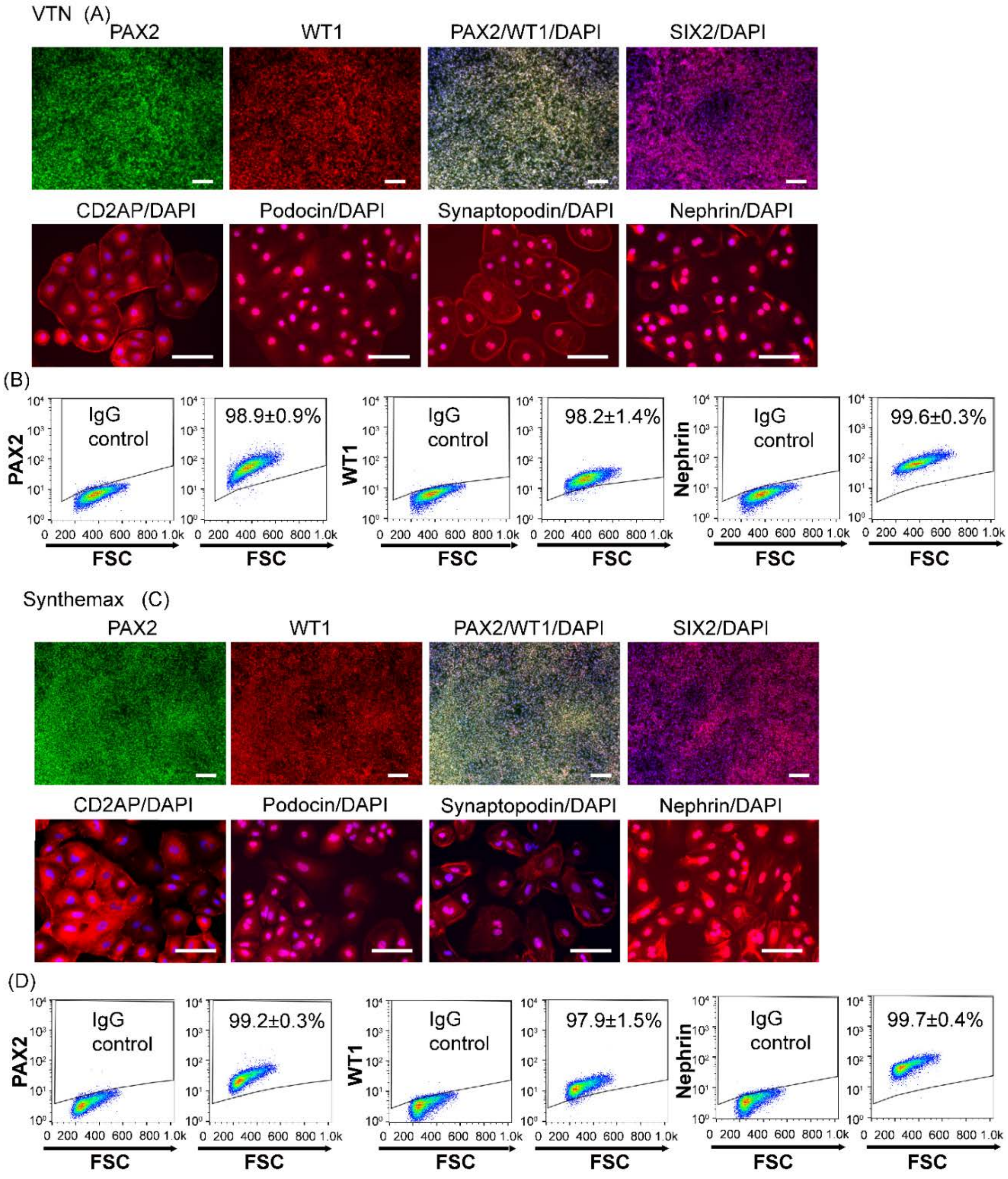

Figure S6. Podocytes differentiated on substrates coated with Synthemax or vitronectin express podocyte-related proteins. Related to Figure 4. Podocytes were differentiated as illustrated in Fig. 1A on surfaces coated with either Synthemax or vitronectin (VTN). At day 6, expression of PAX2, WT1, and SIX2 were verified by immunofluorescence (A, C). At day 16, CD2AP, podocin, synaptopodin, and nephrin were verified by immunofluoresnce (A, C). At day 16, the indicated proteins were detected by flow cytometry (B, D). The numbers on the flow cytometry plots indicate the percentage of cells in the gated region. Data are presented as mean ± SEM of three independent experiments. Scale bars, 100 μm.

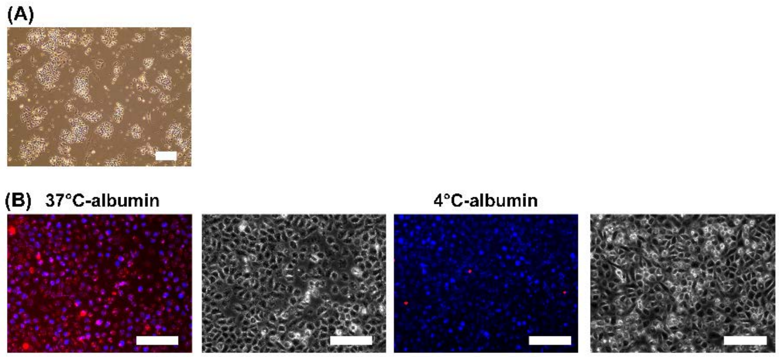

Figure S7. Primary podocytes exhibit cobblestone morphology and albumin uptake ability. (A) Phase contrast image of primary podocytes. (B) Primary podocytes were analyzed with an Albumin Uptake Assay Kit. Alexa Fluor™ 555 -labelled albumin is shown in red on a merged DAPI image and the corresponding bright field image is provided on the right. 4°C was used as a control to prevent endocytosis. Scale bars, 100 µm.

Table S1. Antibodies used in this study. Related to Experimental Procedures.

Antibody	Vendor	Cat. NO.	Fixation	Dilution	Buffer
Brachyury	Abcam	Ab209665	4%PFA	1:200 IF,WB	10% PBSG
PAX2	Santa Cruz	sc-377181	4%PFA	1:200 IF	10% PBSG
WT1	Santa Cruz	sc-192	4% PFA	1:250 IF, WB	10% PBSG
P-cadherin	Santa Cruz	sc-33635	4% PFA	1:100 IF,WB	10% PBSG
Synaptopodin	Santa Cruz	Sc-50459	4% PFA	1:200 IF,WB	10% PBSG
Podocin	Abcam	Ab50339	4% PFA	1:200 IF,WB	10% PBSG
Nephrin	Abcam	Ab72908	4% PFA	1:200 IF	10% PBSG
Nephrin	Abcam	Ab58968		1:500WB	5% milk
Nephrin	Santa Cruz	sc-376522		1:200WB	5% milk
AQP1	Santa Cruz	sc-25287	4% PFA		10% PBSG
CD2AP	Santa Cruz	SC-9137	4% PFA	1:100 IF	10% PBSG
SIX2	Proteintech	11562-1-AP	4% PFA	1:200 IF	10% PBSG
ZO-1	Invitrogen	402200	4% PFA	1:200 IF	10% PBSG
Ki67	BD	550609	4% PFA	1:100 IF	10% PBSG
OCT3/4	Santa Cruz	sc-5279	4% PFA	1:100 IF	10% PBSG
TRA-1-60	Santa Cruz	sc-21705	4% PFA	1:100 IF	10% PBSG
NANOG	Santa Cruz	sc-374001	4% PFA	1:100 IF	10% PBSG
β-actin	Cell signaling	5125s		1:2000WB	5% milk
P-SMAD	Cell signaling	13820		1:1000 WB	5% BSA
SMAD	Cell signaling	6944		1:1000 WB	5% milk
BMP7	Abcam	Ab54904	4% PFA	1:100 IF	10% PBSG
MIXL1	MilliporeSigma	ABS232	4% PFA	1:100 IF	10% PBSG
HOXD11	Santa Cruz	sc-81969	4% PFA	1:50 IF	10% PBSG
Mouse IgG1 κ isotype control	BD	554121		1:100 Flow cytometry	10% PBSG
Rabbit IgG isotype control	BD	550875		1:100 Flow cytometry	10% PBSG
Goat IgG Control	Abcam	Ab37373		1:100 Flow cytometry	1% BSA

Table S2. qPCR primers used in this study. Related to Experimental Procedures

Gene name		Primer length	Product length
GAPDH			207
Forward	CTGATTTGGTCGTATTGGGC	20	
Reverse	TGGAAGATGGTGATGGGATT	20	
POU5F1			120
Forward	GTGGAGGAAGCTGACAACAA	20	
Reverse	ATTCTCCAGGTTGCCTCTCA	20	
MIXL1			130
Forward	GGCGTCAGAGTGGGAAATCC	20	
Reverse	GGCAGGCAGTTCACATCTACC	21	
PAX2			92
Forward	TCAAGTCGAGTCTATCTGCATCC	23	
Reverse	CATGTCACGACCAGTCACAAC	21	
WT1			142
Forward	TCGGCTTACGGGTCGTTG	18	
Reverse	TGAAGGCGCTCAGGCACT	18	
SIX2			247
Forward	AGCGGCAAGTCGGTGTTAG	19	
Reverse	GGTTGGCTGACATGGGGTT	19	
TJP1			128
Forward	ACCAGTAAGTCGTCCTGATCC	21	
Reverse	TCGGCCAAATCTTCTCACTCC	21	
CDH3			121
Forward	TGGAGATCCTTGATGCCAATGA	22	
Reverse	GCGTCCAGATCAGTGACCG	19	
SYNPO			89
Forward	CCGCAAATCCATGTTTACTT	20	
Reverse	GCTTCTCATCCGCTGTCTGT	20	

Western blot full images

1. **Figure 1F brachyury full images**

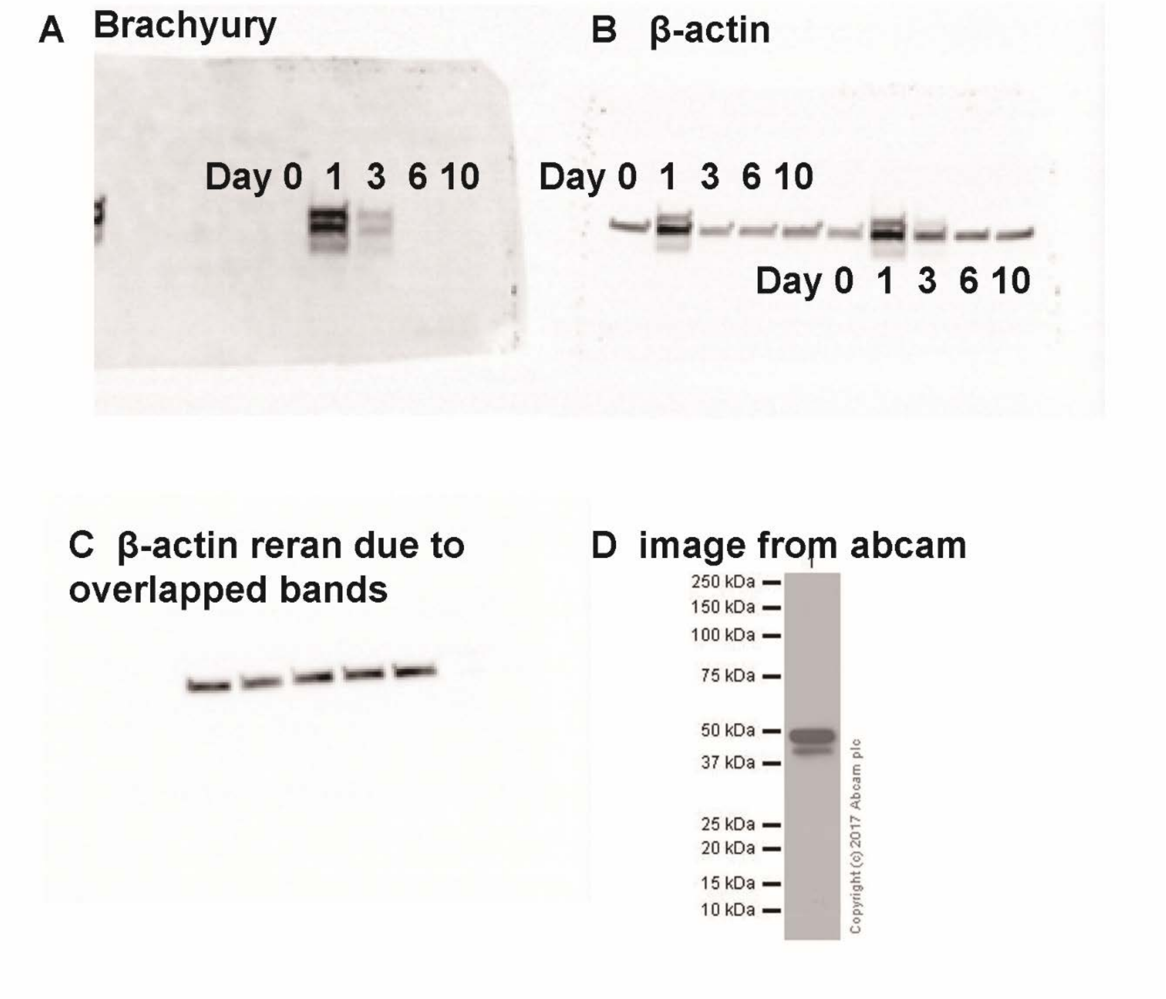

2. Figure 3B WT1 8 conditions

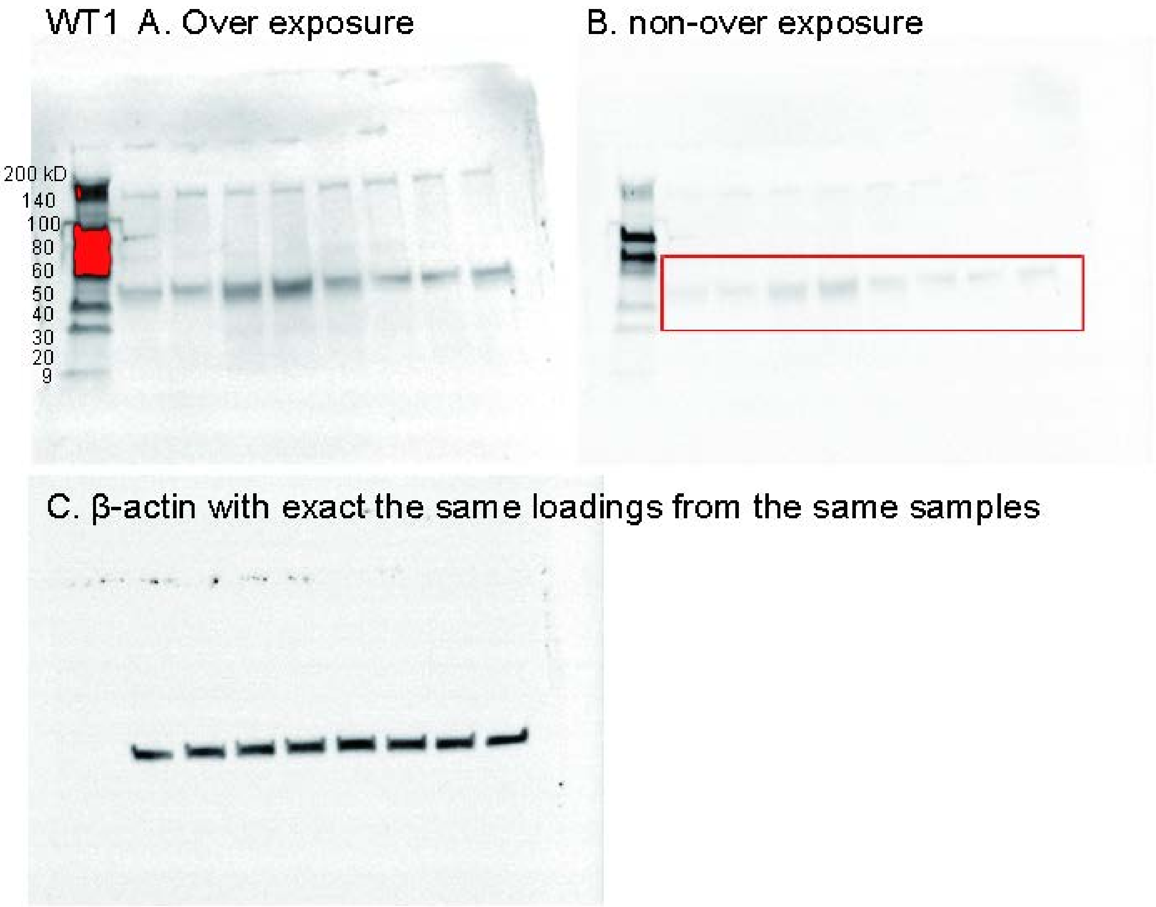

3. **Figure 3D P-SMAD**

P-SMAD Dorsomorphin D1-D3

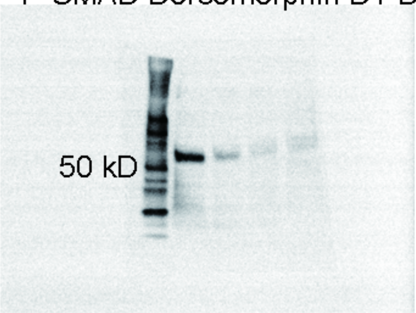

P-SMAD Dorsomorphin D1-D3
β-actin

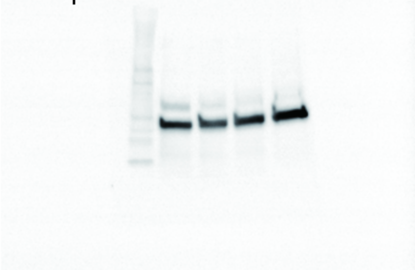

Total SMAD Dorsomorphin D1-D3

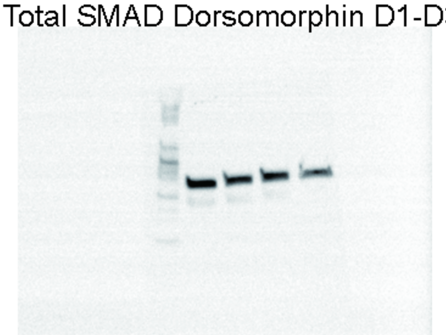

Total SMAD Dorsomorphin D1-D3
β-actin

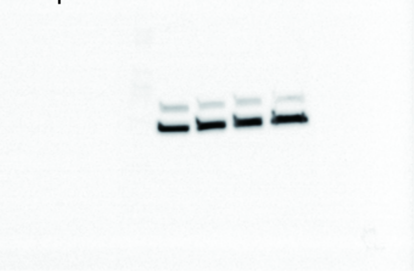

P-SMAD Dorsomorphin D3-D4

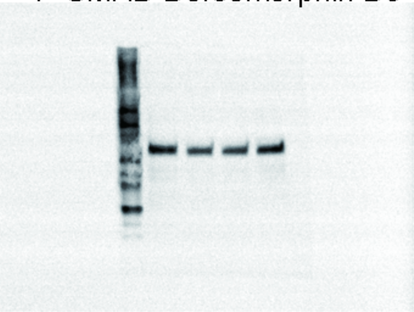

P-SMAD Dorsomorphin D3-D4
β-actin

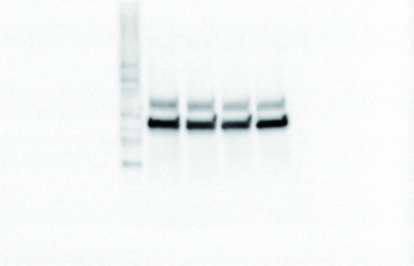

Total SMAD Dorsomorphin D3-D4

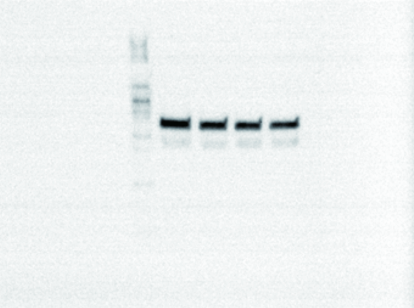

Total SMAD Dorsomorphin D3-D4
β-actin

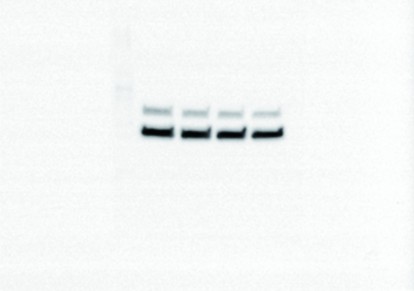

4. **Figure 3G BMP7**

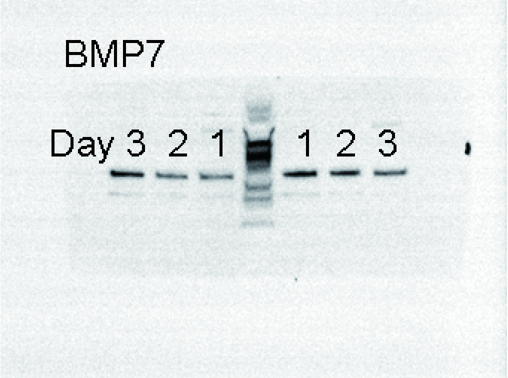

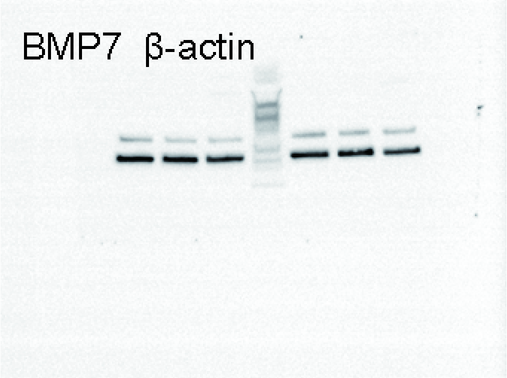

5. **Figure 4 A-D WT1, P-cadherin, Synaptopodin, Nephrin**

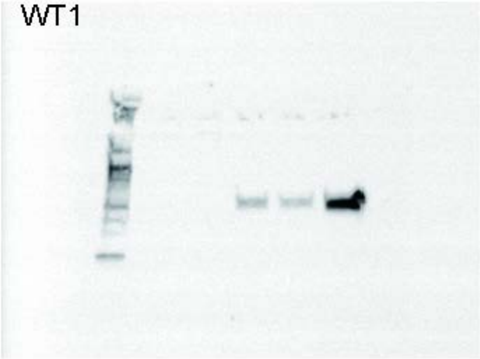

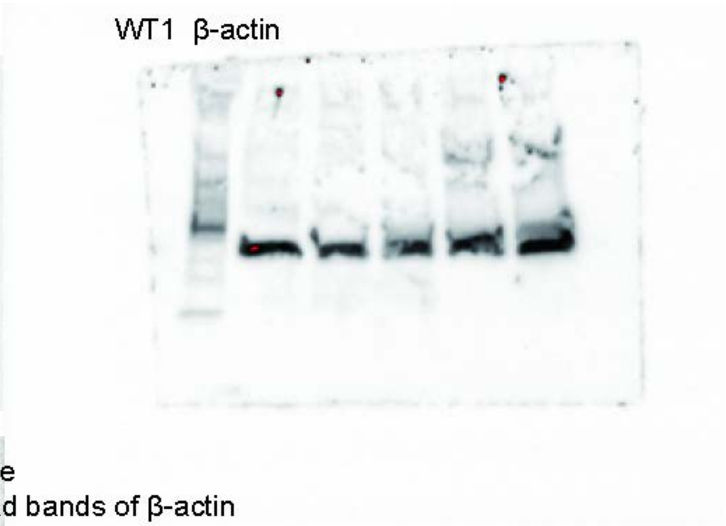

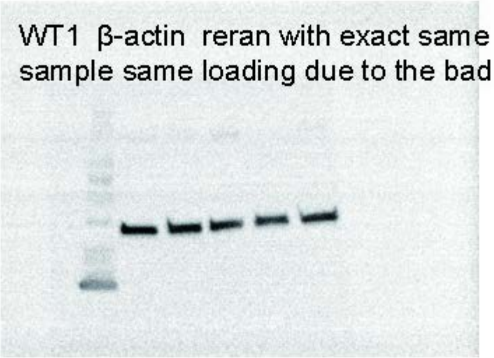

P-cadherin

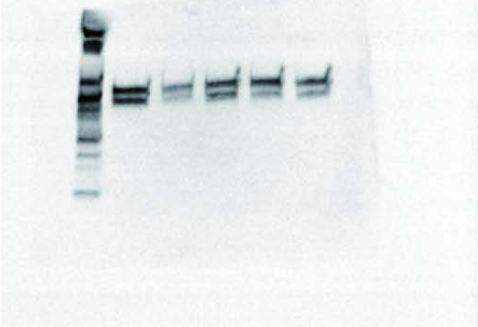

P-cadherin β-actin

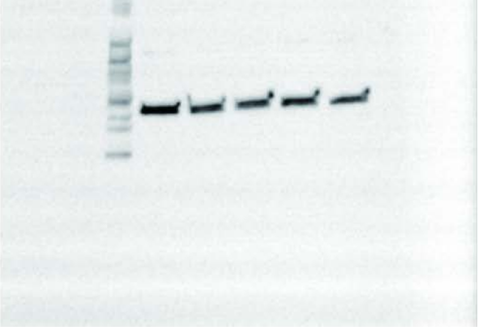

Synaptopodin

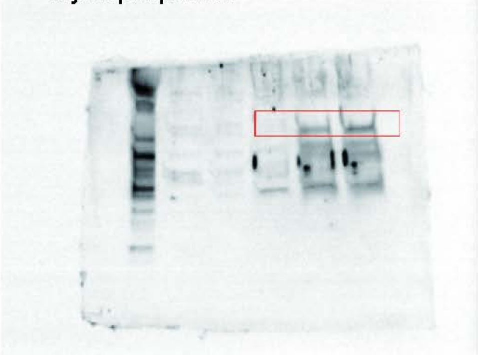

Synaptopodin β-actin

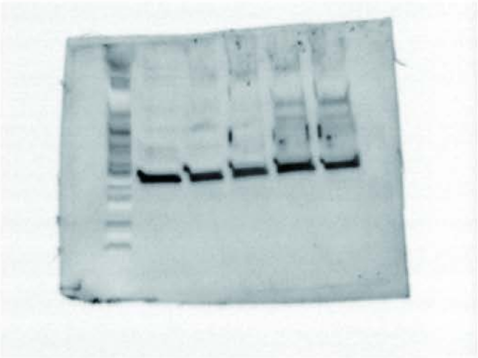

Nephrin

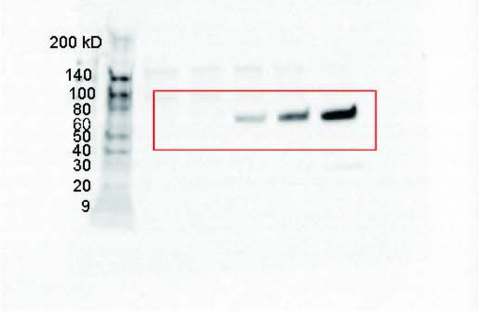

Nephrin β-actin

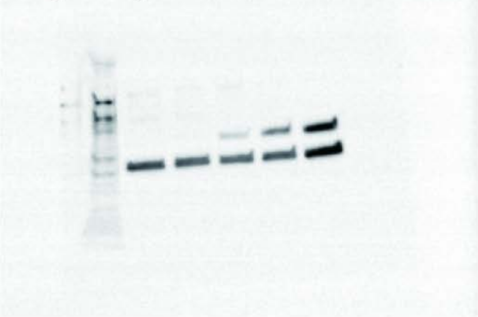

Image from abcam

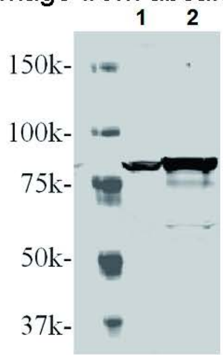

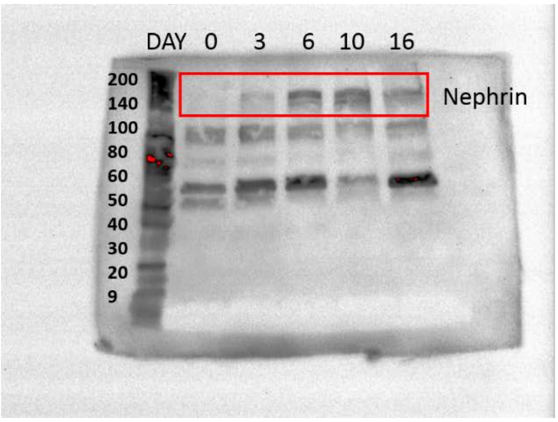
DAY 0 3 6 10 16
200
140
100
80
60
50
40
30
20
9
Nephrin

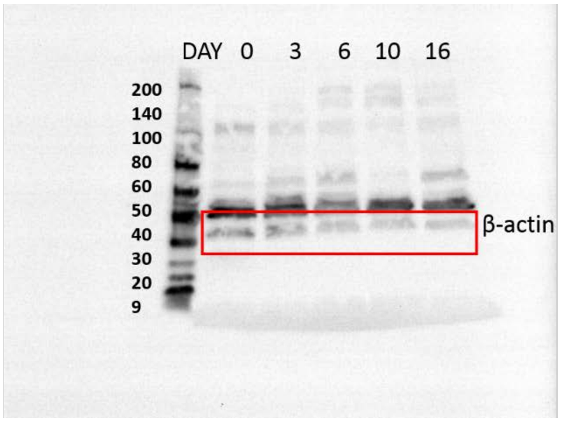
DAY 0 3 6 10 16
200
140
100
80
60
50
40
30
20
9
β-actin
